# The feasibility and surgical outcomes of robotic vaginal natural orifice transluminal endoscopic single port hysterectomy for benign gynecologic diseases: a systematic review and meta-analysis

**DOI:** 10.1016/j.xagr.2025.100512

**Published:** 2025-05-14

**Authors:** Greg Marchand, Hollie Ulibarri, Amanda Arroyo, Daniela Gonzalez Herrera, Brooke Hamilton, Kate Ruffley, Mckenna Robinson, Ali Azadi

**Affiliations:** 1 Marchand Institute for Minimally Invasive Surgery, Mesa, AZ (Marchand, Ulibarri, Arroyo, Herrera, Hamilton, Ruffley, and Robinson); 2 College of Medicine, University of Arizona, Phoenix, AZ (Azadi); 3 School of Medicine, Creighton University, Phoenix, AZ (Azadi)

**Keywords:** hysterectomy, laparoscopy, RALH, robotic-assisted, R-vNOTES, vNOTES

## Abstract

**Objective:**

Vaginal natural orifice transluminal endoscopic surgery (vNOTES) combines vaginal surgery with single-port laparoscopy, providing a minimally invasive technique designed to overcome the challenges in traditional vaginal surgery. Several authors have now described techniques for performing these procedures with robotic assistance (R-vNOTES). We aim to evaluate the surgical outcomes and the safety of R-vNOTES hysterectomy in patients with benign diseases.

**Data Sources:**

We searched six major databases from their inception through October 2024 for studies analyzing the surgical outcomes of hysterectomy by R-vNOTES in cases with benign gynecologic diseases.

**Study eligibility criteria:**

We included all primary research studies that included at least one of our selected outcomes and did not include surgeries for malignant conditions.

**Study appraisal and synthesis methods:**

Study quality was appraised using the National Heart, Lung, and Blood Institute quality assessment tools. Data synthesis was accomplished using OpenMetaAnalyst and RevMan software. Mean difference and 95% confidence intervals were used for continuous outcomes following inverse variance analyses. Dichotomous outcomes were analyzed using an odds ratio and 95% confidence intervals.

**Results:**

Ultimately 10 eligible studies were included in our synthesis, including two studies that compared the R-vNOTES technique to robot-assisted laparoscopic hysterectomy (RALH) performed for the same indications. Our overall pooled analysis demonstrated that the operation time of R-vNOTES was 142 minutes, with an estimated blood loss of 67 mL. The overall length of hospital stay among the included studies was 2.04 days. We found an approximate decrease of 1.4 grams of hemoglobin after surgery. The incidence of conversion was 1.3%, and the complication rate was 13.3%. We found, R-vNOTES was to have a shorter operative time (*P*<.001) and lower blood loss than RALH (*P*=.002), with no significant differences seen between the cohorts in total hospital stay (*P*=.29) or complication rates (*P*=.98).

**Conclusion:**

Initial data shows that R-vNOTES seems to be a feasible minimally invasive technique with comparable outcomes and a favorable safety profile. Compared to RALH, R-vNOTES was associated with a shorter operation time and less blood loss.

**AJOG Global Reports at a Glance**

**Why was this study conducted?**

Robotic Vaginal Natural Orifice Transluminal Endoscopic Single Port laparoscopy is a recently described technique, and much interest surrounds whether this technique is a safe and feasible method of performing hysterectomy for benign diseases.

**Key findings**

In comparing robotic vaginal natural orifice transluminal endoscopic single port hysterectomy to robotic laparoscopic hysterectomy, most safety and efficacy outcomes were comparable, with a lower operative time and less blood loss in the natural orifice group.

**What does this add to what is known?**

This study begins the process of looking at the new technique as it gains momentum in minimally invasive gynecology. The initial evidence from this analysis speaks to this technique as seeming to be safe and feasible, with a need for further research in this area.

## Introduction

Hysterectomy, with over 500,000 procedures annually in the United States, is a common gynecologic surgery for benign conditions like fibroids, prolapse, or abnormal bleeding.^1^^,^^2^ Traditional approaches include abdominal, laparoscopic, and vaginal hysterectomy, but new minimally invasive techniques aim to reduce operative time, blood loss, and recovery duration.^3^, ^4^, ^5^ Vaginal natural orifice transluminal endoscopic surgery (vNOTES) combines vaginal surgery with single-port laparoscopy to address challenges of traditional vaginal surgery, though it faces issues like instrument crowding.^6^, ^7^, ^8^, ^9^ Specifically, vNOTES involves laparoscopic surgery through the vaginal orifice using a single-port device to access the pelvic cavity without abdominal incisions. Robotic-assisted vNOTES (R-vNOTES) enhances this technique with robotic systems, providing three-dimensional visualization and flexible, wristed instruments for improved precision and control.^7^^,^^10^, ^11^, ^12^, ^13^, ^14^ R-vNOTES enhances this approach with improved 3D visualization and flexible instruments.^15^^,^^16^ This study evaluates the surgical outcomes and safety of R-vNOTES hysterectomy for benign gynecologic diseases.

## Methods

We adhered to the recommendations contained within the PRISMA guidelines while conducting this study,^17^ and have completed PRISMA checklists and composed a flow chart of our literature search process.

### Search and information databases

We implemented the following strategy in performing our search, which encompassed all results from each search engine’s inception until October 1, 2024: (Robotic-assisted vaginal natural orifice transluminal endoscopic surgery OR robotic NOTES OR robotic vNOTES) AND (robot-assisted laparoscopic OR robotic-assisted laparoscopy OR robotic-assisted single-site port) AND hysterectomy. Scopus, ClinicalTrials.Gov, Medline, Web of Science, PubMed, and Cochrane Library were the searched databases.

### Selection criteria and eligibility criteria

We carried out the selection process of the eligible studies in two stages: the initial stage was the screening of titles and abstracts to find relevant studies. Both stages were carried out in duplicate by two researchers. Any discrepancies were handled by consensus. A third researcher was assigned to settle any disputes, but ultimately none arose. The second stage was the full-text screening of the articles which were selected from the first stage according to the following criteria:

**Population:** Women with benign diseases requiring hysterectomy.
**Intervention:** Hysterectomy by R-vNOTES.
**Comparator:** Any or no comparator (single arm), and specifically those comparing R-vNOTES with robot-assisted laparoscopic hysterectomy (RALH).
**Outcomes:** Total operative time, surgeon declared estimated blood loss (EBL), length of hospitalization (in days), the change in hemoglobin levels, the incidence of complications, the incidence of conversion to other techniques, pain scores after 24 hours, and pain scores after 1, 2, and 3 weeks.
**Study design**: We excluded studies that included patients with malignant diseases, articles that did not assess our outcomes, and secondary research such as systematic reviews and meta-analyses.

### Data extraction

We collected the general characteristics from the included studies, such as study design, age, BMI, parity, the number of prior surgeries, and the uterine weight. In addition, we extracted data for all of the hysterectomy indications. We also collected data of the primary outcomes, including length of surgery (in minutes), EBL (in mL), length of hospitalization (in days), postoperative hemoglobin drop (in g/dL), the incidence of complications (author defined), the incidence of conversion to other techniques, pain scores after 24 hours, 1 week, 2 weeks, and 3 weeks.

### Quality assessment

Ultimately only observational studies were included in our analysis. Thus, we employed the National Heart, Lung, and Blood Institute (NHLB) quality assessment tools for evaluating the risk of bias in these studies.^18^

### Statistical analysis

The collected data were analyzed using OpenMetaAnalyst and RevMan software. Mean difference (MD) and 95% confidence intervals were employed to analyze continuous outcomes following the inverse variance analysis. Concerning the dichotomous outcomes, they were analyzed using an odds ratio (OR) and 95% confidence intervals. The heterogeneity among the analyzed studies was measured using the Chi-square test *P* value and the *I*^2^ value. The outcome was considered heterogeneous if *I*^2^ > 50% or *P*<.1.^19^

## Results

### Summary of the included studies

**Figure 1:**
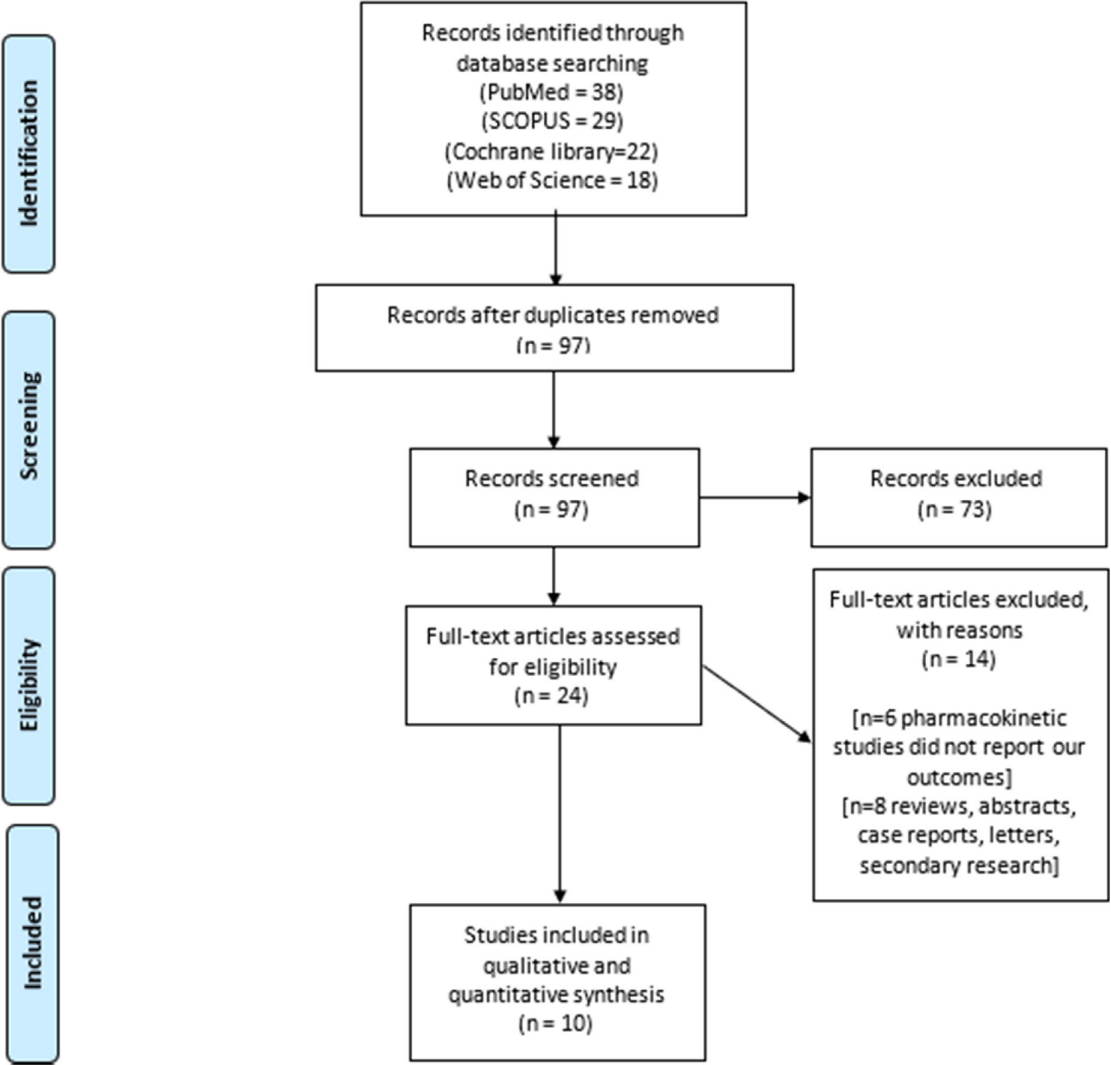
PRISMA flowchart of our literature search process

We described our search results in the PRISMA flow diagram (Figure 1). Ultimately, we included 10 studies^7^^,^^20^, ^21^, ^22^, ^23^, ^24^, ^25^, ^26^, ^27^, ^28^ into our final synthesis. This combined a total of 432 surgical cases who underwent R-vNOTES hysterectomy and 286 cases from two studies^21^^,^^25^ who underwent RALH. The baseline characteristics and indications of surgery are illustrated in Tables 1 and 2.

Marchand. The feasibility and surgical outcomes of robotic vaginal natural orifice transluminal endoscopic single port hysterectomy for benign gynecologic diseases. AJOG Glob Rep 2025.

**Table 1:** Baseline characteristics of the included patients

		Sample size	Age, y (mean±SD)	BMI (kg/m^2^)	Parity (mean±SD)	Prior surgery, *n* (%)	Uterine weight (gm)
Study	Study design	R-vNOTES	R-vNOTES	R-vNOTES	R-vNOTES	R-vNOTES	R-vNOTES
Imai et al^21^	Retrospective study	24	48.25±7.44	23.47±3.67	NR	4 (28.6)	254.25±117.36
Koythong et al^7^	Retrospective case series	35	41.5±1.9	28.75±2.13	NR	2 (5.7)	136±115.94
Lee et al^22^	Retrospective study	4	45.5±2.5	25.5±1.2	1.0±0.6	NR	365.5±138.4
Lowenstein et al^24^	Prospective study	15	58.5±12.07	27.1±6.43	3.5±2.87	12 (40)	NR
Liu et al^23^	Retrospective study	84	44.5±9.03	30.18±6.03	2.25±1.026	65 (77.3)	433.25±283.21
Thigpen et al^25^	Retrospective study	159	40.66±6.73	29±8.23	NR	2 (1.25)	151.3±130.17
Guan et al^20^	Retrospective case series	28	40.1±7.2	28.5±7.2	NR	21 (75)	NR
Xu et al^26^	Retrospective study	37	47.43±4.44	23.16±2.72	NR	11 (29.73)	NR
Yang^27^	Retrospective study	13	46.7±4.8	24.1±3.5	1.75±0.89	5 (38.5)	396.75±232.22
Zhang et al^28^	Retrospective case series	33	39.27±5.37	27.36±6.51	NR	NR	NR

Data are reported as mean±standard deviation or number (percentage).

*BMI*, body mass index; *NR*, not reported; *R-vNOTES*, robotic vaginal natural orifice transluminal endoscopic surgery; *SD*, standard deviation.

**Table 2:** Indications for hysterectomy among the included studies

	Indication for surgery, *n* (%)				
	Myoma	Endometrial hyperplasia	Bleeding	Adnexal mass	Adenomyosis
Study	R-vNOTES	R-vNOTES	R-vNOTES	R-vNOTES	R-vNOTES
Imai et al^21^	17 (70.8)	1 (4.2)	NR	NR	1 (4.2)
Koythong et al^7^	NR	NR	NR	NR	NR
Lee et al^22^	3 (75)	NR	NR	NR	1 (25)
Lowenstein et al^24^	4 (13)	2 (7)	7 (23)	2 (7)	0
Liu et al^23^	11 (13.1)	NR	NR	NR	3 (3.57)
Thigpen et al^25^	NR	NR	NR	NR	NR
Guan et al^20^	1 (3.6)	NR	4 (14.3)	NR	NR
Xu et al^26^	10 (27.03)	1 (2.7)	NR	NR	11 (29.73)
Yang^27^	6 (42.9)	0	0	0	6 (42.9)
Zhang et al^28^	6 (18.18)	NR	7 (21.2)	NR	NR

Data are reported as number (percentage).

*NR*, not reported; *R-vNOTES*, robotic vaginal natural orifice transluminal endoscopic surgery.

### Results of risk of bias assessment

We utilized the NHLB quality assessment tool to assess the risk of bias in our included studies. The mean quality assessment score of the observation cohort studies score was 9.57 out of 14. In comparison, the average score of the included case series studies was 8.6 out of 9, as shown in Tables 3 and 4.^18^

**Table 3:** Quality assessment of the included observational and cohort studies

	Imai et al^21^	Lee et al^22^	Lowenstein et al^24^	Liu et al^23^	Thigpen et al^25^	Xu et al^26^	Yang^27^
1. Was the research question or objective in this paper clearly stated?	1	1	1	1	0	1	1
2. Was the study population clearly specified and defined?	1	1	1	1	1	1	1
3. Was the participation rate of eligible persons at least 50%?	1	1	1	1	1	1	1
4. Were all the subjects selected or recruited from the same or similar populations (including the same time period)? Were inclusion and exclusion criteria for being in the study prespecified and applied uniformly to all participants?	0	1	1	1	1	1	0
5. Was a sample size justification, power description, or variance and effect estimates provided?	0	0	0	0	0	0	0
6. For the analyses in this paper, were the exposure(s) of interest measured prior to the outcome(s) being measured?	1	1	1	1	1	1	1
7. Was the timeframe sufficient so that one could reasonably expect to see an association between exposure and outcome if it existed?	0	1	1	1	1	1	0
8. For exposures that can vary in amount or level, did the study examine different levels of the exposure as related to the outcome (eg, categories of exposure, or exposure measured as continuous variable)?	0	0	0	0	0	0	0
9. Were the exposure measures (independent variables) clearly defined, valid, reliable, and implemented consistently across all study participants?	1	1	1	1	1	1	1
10. Was the exposure(s) assessed more than once over time?	1	1	1	1	1	1	1
11. Were the outcome measures (dependent variables) clearly defined, valid, reliable, and implemented consistently across all study participants?	1	1	1	1	1	1	1
12. Were the outcome assessors blinded to the exposure status of participants?	*	*	*	*	*	*	*
13. Was loss to follow-up after baseline 20% or less?	1	0	1	1	*	1	1
14. Were key potential confounding variables measured and adjusted statistically for their impact on the relationship between exposure(s) and outcome(s)?	1	0	0	1	1	0	1
Total score (out of 14)	9/14	9/14	10/14	11/14	9/14	10/14	9/14

Key: 1 = yes, 0 = no, * = not reported, n/a = not applicable.

**Table 4:** Quality assessment of the included case series studies

	Koythong et al^7^	Guan et al^20^	Zhang et al^28^
1. Was the research question or objective in this paper clearly stated?	1	1	1
2. Was the study population clearly and fully described, including a case definition?	1	1	1
3. Were the cases consecutive?	1	1	1
4. Were the subjects comparable?	1	1	1
5. Was the intervention clearly described?	1	1	1
6. Were the outcome measures clearly defined, valid, reliable, and implemented consistently across all study participants?	1	1	1
7. Was the length of follow-up adequate?	1	1	0
8. Were the statistical methods well-described?	1	1	1
9. Were the results well-described?	1	1	1
Total score (out of 9)	9/9	9/9	8/9

Key: 1 = yes, 0 = no, * = not reported, n/a = not applicable.

### Analysis of outcomes

#### Operative time

All studies reported operative time for R-vNOTES.^7^^,^^20^, ^21^, ^22^, ^23^, ^24^, ^25^, ^26^, ^27^, ^28^ The pooled operative time was 142 minutes (95% CI: 115–169, OR: 115–169, *P*<.001), ranging from 56 minutes (Lowenstein et al) to 235 minutes (Guan et al). Heterogeneity was noted, likely due to variations in patient populations or surgical experience (Supplemental Figure S1)*.* See Table 1 for study-specific details.

**Figure 2:**
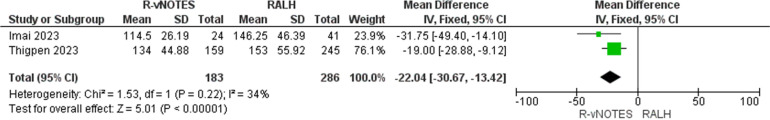
Forest plot for the meta-analysis of total operative time for the studies comparing the R-vNOTES and RALH techniques

Two studies^21^^,^^25^ compared the operative time between the R-vNOTES technique and RALH. The combined analysis showed a significantly shorter operation time in the R-vNOTES cohort (MD=–22.04 [–30.67, –13.42], (*P*<.001). The analysis was homogeneous (Figure 2).

Marchand. The feasibility and surgical outcomes of robotic vaginal natural orifice transluminal endoscopic single port hysterectomy for benign gynecologic diseases. AJOG Glob Rep 2025.

#### Estimated blood loss

All studies reported EBL for R-vNOTES.^7^^,^^20^, ^21^, ^22^, ^23^, ^24^, ^25^, ^26^, ^27^, ^28^ The pooled EBL was 67 mL (95% CI: 55–80, *P*<.001), ranging from 24 mL (Xu et al) to 180 mL (Lee et al). Heterogeneity was observed, possibly due to surgical or patient variability (Supplemental Figure S2). See Table 1 for study-specific EBL data.

**Figure 3:**
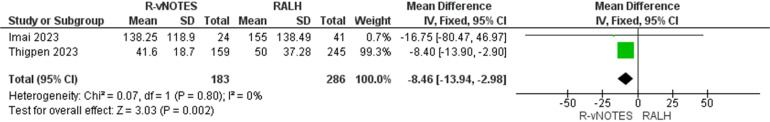
Forest plot for the meta-analysis of the surgeon declared estimated blood loss (in mL,) for the studies comparing the R-vNOTES and RALH techniques

Imai et al and Thigpen et al^21^^,^^25^ also compared R-vNOTES and RALH regarding the intraoperative EBL. The combined MD showed that women who underwent R-vNOTES experienced significantly lower blood loss by MD of 8 mL [–14, –3] (*P*=.002). There was low heterogeneity in this analysis (*P*=.80); *I*²=0% (Figure 3).

Marchand. The feasibility and surgical outcomes of robotic vaginal natural orifice transluminal endoscopic single port hysterectomy for benign gynecologic diseases. AJOG Glob Rep 2025.

#### Length of hospital stay

Nine studies reported length of hospital stay for R-vNOTES.^7^^,^^21^, ^22^, ^23^, ^24^, ^25^, ^26^, ^27^, ^28^ The pooled estimate was 2 days (95% CI: 1–3, *P*<.001), ranging from 0.3 days (Thigpen et al, Koythong et al) to 4 days (Lowenstein et al). Heterogeneity was noted, likely due to institutional differences (Supplemental Figure S3). See Table 1 for study-specific data.

**Figure 4:**
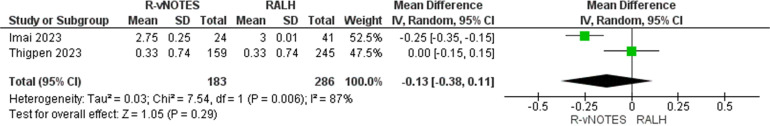
Forest plot for the meta-analysis of the total length of hospital stay (in days,) for the studies comparing the R-vNOTES and RALH techniques

The pooled analysis of both studies comparing R-vNOTES and RALH revealed a similar length of hospitalization in both groups (MD=–0.13 [–0.38, 0.11], (*P*=.29). Heterogeneity was observed among the data (*P*=.006); *I*²=87% (Figure 4).

Marchand. The feasibility and surgical outcomes of robotic vaginal natural orifice transluminal endoscopic single port hysterectomy for benign gynecologic diseases. AJOG Glob Rep 2025.

### Complications

Eight studies^7^^,^^20^^,^^21^^,^^23^^,^^25^, ^26^, ^27^, ^28^ assessed the incidence of complications in women who underwent R-vNOTES. In order to maintain data compatibility, we accepted the definition of a complication from each study, although essentially all studies accepted a complication as a serious unexpected result of surgery in the 90 days following the procedure. The complication rates varied from 0% to 17.1%. Imai et al reported the lowest proportion at 4.2% (1/24), while Koythong et al had the highest at 17.1% (6/35). Similarly, Liu et al and Zhang et al had complication rates of 15.5% (13/84) and 15.2% (5/33), respectively. Thigpen et al observed a rate of 16.4% (26/159). Guan et al and Xu et al observed rates of 7.1% (2/28) and 5.4% (2/37), respectively. Yang et al reported 0% complications (0/13). The pooled incidence of complications was 13.3% (55/413), with an overall proportion of 0.104 [0.061, 0.147] (*P*<.001) (Supplemental Figure S4).

**Figure 5:**
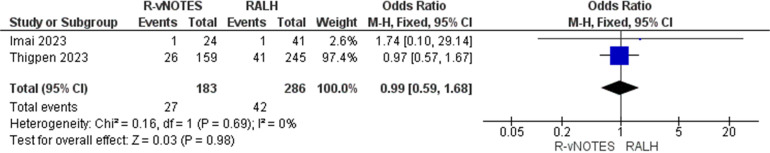
Forest plot for the meta-analysis of the complication rate for the studies comparing the R-vNOTES and RALH techniques

The combined OR of both studies comparing R-vNOTES and RALH revealed a comparable incidence of complications in both cohorts (OR=0.99 [0.59, 1.68], (*P*=.98)). We found no heterogeneity among studies (*P*=.69); *I*²=0% (Figure 5).

Marchand. The feasibility and surgical outcomes of robotic vaginal natural orifice transluminal endoscopic single port hysterectomy for benign gynecologic diseases. AJOG Glob Rep 2025.

### Pain score

The postoperative pain score, measured using the Visual Analog Scale after 24 hours was evaluated by three studies.^24^^,^^26^^,^^27^ Yang et al reported the lowest pain score of 1.25, followed by Lowenstein et al with a pain score of 3. In contrast, the highest pain score after 24 hours was reported by Xu et al at 5.3. The estimated pain score after 24 hours was 3.206 [0.861, 5.552] (*P*=.007) (Supplemental Figure S5). Regarding the pain scores after the first 3 weeks, Liu et al reported the highest pain score of 6.85 in the first week, which decreased to 4.83 and 2.83 after the second and the third weeks, respectively. Zhang et al reported a pain score of 6.73 in the first week that improved to 4.81 and 2.63 after the second and the third weeks, respectively. Koythong et al demonstrated that the first-week pain score was 6, which showed a dramatic decrease in the second and third weeks to 4 and 2, respectively. Thigpen et al reported the lowest pain scores among all studies, with 5.83 in the first week, which improved to 3.5 and 1.66 in the second and third weeks, respectively. Guan et al reported a pain score of 4.7 in the first week, which improved to 3.3 and 2.1 in the second and third weeks, respectively. The combined analysis of the pain scores in all studies^7^^,^^20^^,^^23^^,^^25^^,^^28^ after the first week, second week, and third week showed estimates of 6.095 [5.352, 6.837], 4.112 [3.331, 4.892], and 2.260 [1.593, 2.926], (*P*<.001), respectively (Supplemental Figures S6–S8). Notably, scores >6 at 1 week and ∼4 at 2 weeks suggest suboptimal pain relief, as scores <3.1 typically indicate adequate pain control. This may reflect variability in pain management protocols or patient reporting and warrants further investigation.

### Change in hemoglobin levels

Four studies reported the postoperative hemoglobin change.^21^^,^^22^^,^^24^^,^^27^ Imai et al reported the most significant decrease in hemoglobin postoperatively, showing a mean reduction of 2 g/dL. Lee et al and Lowenstein et al observed a moderate postoperative hemoglobin reduction of 1.6 g/dL and 1.4, respectively. Yang et al reported the smallest decrease in hemoglobin levels, with a mean reduction of 0.67 g/dL. Pooled analysis showed an overall postoperative hemoglobin reduction of –1.415 g/dL [–1.952, –0.879] (*P*<.001) (Supplemental Figure S9).

### Conversion

Seven studies reported the incidence of conversion to other surgical techniques.^7^^,^^20^^,^^23^^,^^25^, ^26^, ^27^, ^28^ The conversion rate ranged from 0% to 3.6%, with Guan et al reporting the highest conversion rate (3.6%) followed by Zhang et al with 3.0%. Liu et al reported a conversion rate of 2.4%. Thigpen et al reported a low incidence of conversion rate with 0.6%. While Koythong et al, Lowenstein et al, and Yang, reported no conversions. We found a total of 5 conversions out of 367 operations (1.3%), with an overall proportion of 0.011 [0.000, 0.021] (*P*=.043) (Supplemental Figure S10).

## Discussion

In recent decades, there have been considerable innovations in the development of surgical techniques. Conventional open laparotomy has been largely substituted with minimally invasive procedures such as laparoscopic surgery, single-port laparoscopic surgery, robotic-assisted laparoscopy, and v-NOTES.^29^^,^^30^ This occurred due to the recent concepts that aim to improve perioperative management, clinical outcomes, rehabilitation, and patient quality of life. Previous evidence showed that vaginal hysterectomy was superior to abdominal open surgery in terms of shorter operation time, fewer intraoperative complications, shorter hospitalization, and quicker recovery.^31^ Thus, the American College of Obstetricians and Gynecologists has endorsed the vaginal approach as the primary choice for hysterectomy for benign diseases.^32^ However, the frequency of transvaginal hysterectomy has reduced from 25% of patients in 1998 reaching about 17% of patients in 2010 in favor of other minimally invasive techniques such as laparoscopic or robotic-assisted laparoscopy.^33^ Despite the argued superiority, pure vaginal hysterectomy is not a popular technique among most gynecologic surgeons.^33^

This may be secondary to many challenges in vaginal hysterectomy, including the narrow opening, the need for more surgical experience and training, the existence of large masses, severe adhesive disease, and limited uterine descent. Several authors have described that vNOTES may reduce many of these barriers.^34^ vNOTES is performed by the application of laparoscopic techniques through the vaginal orifice, with the potential of providing enhanced visualization and better management of undescended structures. Additionally, vNOTES may be associated with less hematoma formation, fewer wounds, fewer infections, and better hemostasis.^35^ Previous studies have reported the safety and feasibility of vNOTES in hysterectomy, sacrocolpopexy, and adnexal surgeries.^11^^,^^36^ The present study aims to evaluate the surgical outcomes and the safety of R-vNOTES hysterectomy in managing patients with benign diseases.

Robotic-assisted surgery is the most recent computer-enhanced technological advancement in the field of minimally invasive procedures. Robotic-assisted laparoscopic techniques may provide better 3D visualization of the surgery field and a stable wide range of motion, potentially allowing accurate and meticulous dissection and coagulation, and reducing intraoperative complications.

One of the first studies to examine the feasibility of R-vNOTES compared to traditional vNOTES hysterectomy was a retrospective case series, which we have included in the present analysis.^7^ They found similar surgical time, blood loss, duration of hospitalization, and pain scores in both techniques. They reported a 17.1% incidence of complications in R-vNOTES, which is close to our findings, which reached 13.3%. In our study, we also demonstrated a low conversion rate to other surgical procedures (1.3%), reflecting the effectiveness and success of the R-vNOTES procedure. This is similar to previous studies, most of which reported no conversions at all.^7^^,^^24^^,^^27^ This low conversion rate may be attributed to the reduced difficulty and complexity of surgery utilizing robotic instruments, which allow more multi-directional movements when compared to traditional laparoscopy.^23^ However studies that have higher conversion rates have subjectively blamed these events on difficulties arising from poor landmark visualization, working in the narrow vaginal orifice, or visualization obstructed by the presence of a mass.^11^^,^^37^ Therefore, the conversion rate may be significantly decreased by the proper preoperative evaluation of these causes and adequate selection of patients undergoing v-NOTES.^11^^,^^37^

Postoperative pain levels are of particular interest to many surgeons, especially in considering whether a purely vaginal approach is inherently less painful. In our study, we reported that the estimated pain score after 24 hours was 3.206, and the combined pain scores after the first week, second week, and third week showed estimates of 6.095, 4.112, and 2.260 (*P*<.001), respectively. These findings are consistent with previous studies.^7^^,^^23^^,^^25^^,^^28^

In our study, we also investigated R-vNOTES compared to RALH. Our analysis demonstrated that R-vNOTES was associated with shorter operation time and less blood loss, though the 8 mL difference in EBL, while statistically significant (*P*=.002), is likely of limited clinical relevance due to its small magnitude. Notably, the reported hemoglobin drop of 1.4 g/dL appears disproportionate to the low EBL (eg, 8 mL in R-vNOTES vs. RALH), suggesting potential underestimation of blood loss, a known limitation of surgeon-reported EBL. Future studies may consider employing objective measures, such as serial hemoglobin assessments, to better quantify blood loss and avoid underestimation by the surgeon. However, we found no considerable variation between both cohorts regarding the duration of hospitalization and incidence of complications. A previous systematic review comparing the traditional vNOTES with classic laparoscopic hysterectomy found shorter operation time, less hospital stay and better postoperative pain scores in the vNOTES group.^38^ Imai et al described the surgical outcomes and quality of life of women who underwent hysterectomy by R-vNOTES versus RALH. They reported no differences between both techniques in terms of operation time, blood loss, postoperative complications, and uterine weight. However, R-vNOTES was associated with a higher quality of life.^21^ Another retrospective study of 404 patients underwent hysterectomy by R-vNOTES or robotic-assisted single-site port. They demonstrated a significantly shorter operative time in R-vNOTES, which is consistent with our findings. There were no differences regarding the surgical or clinical outcomes except for the postoperative pain score, which was lower in the R-vNOTES hysterectomy cohort.^25^

## Limitations

The heterogeneity observed in some outcomes indicated variability among the analyzed studies. The retrospective nature of included studies, the relatively small sample size, and the lack of double arm analysis due to insufficient published articles comparing R-vNOTES with RALH.

## Conclusion

Our meta-analysis suggests that R-vNOTES is a feasible and reliable minimally invasive technique with favorable outcomes and safety profile. Compared to RALH, R-vNOTES was associated with shorter operation time and less blood loss. However, we found no considerable variation between both cohorts regarding the duration of hospitalization and incidence of complications. Future research, including a larger sample size and standardized reporting of surgical outcomes and complications with long-term follow-up, is required before establishing R-vNOTES as a first-line hysterectomy technique in cases of benign diseases.

## CRediT authorship contribution statement

**Greg Marchand:** Writing – review & editing, Writing – original draft, Supervision, Methodology, Formal analysis. **Hollie Ulibarri:** Formal analysis, Data curation. **Amanda Arroyo:** Validation, Formal analysis, Data curation. **Daniela Gonzalez Herrera:** Methodology, Data curation. **Brooke Hamilton:** Formal analysis, Data curation. **Kate Ruffley:** Formal analysis, Data curation. **Mckenna Robinson:** Funding acquisition, Data curation. **Ali Azadi:** Writing – review & editing, Supervision.

## Appendix Supplementary materials

Image, application 1

## Footnotes

Supplementary material associated with this article can be found in the online version at doi:10.1016/j.xagr.2025.100512.
