# Supplementary material for: The feasibility and surgical outcomes of robotic vaginal natural orifice transluminal endoscopic single port hysterectomy for benign gynecologic diseases: a systematic review and meta-analysis
Source: AJOG Glob Rep. 2025 May 14;5(3):100512. doi: 10.1016/j.xagr.2025.100512 (PMC12182362; doi:10.1016/j.xagr.2025.100512)
Supplement: Supplementary file 1 [file mmc1.docx]

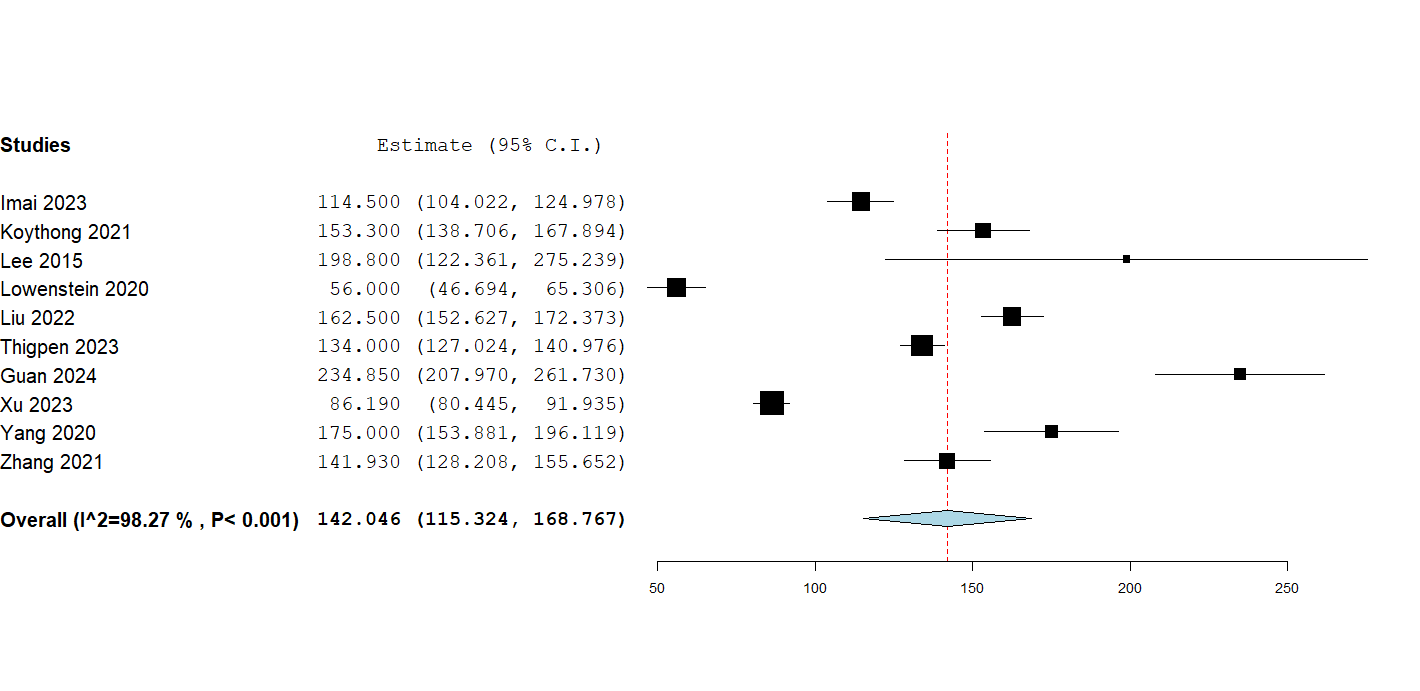


**Supplemental Figure S1:** Forest plot for the meta analysis of total operative time.


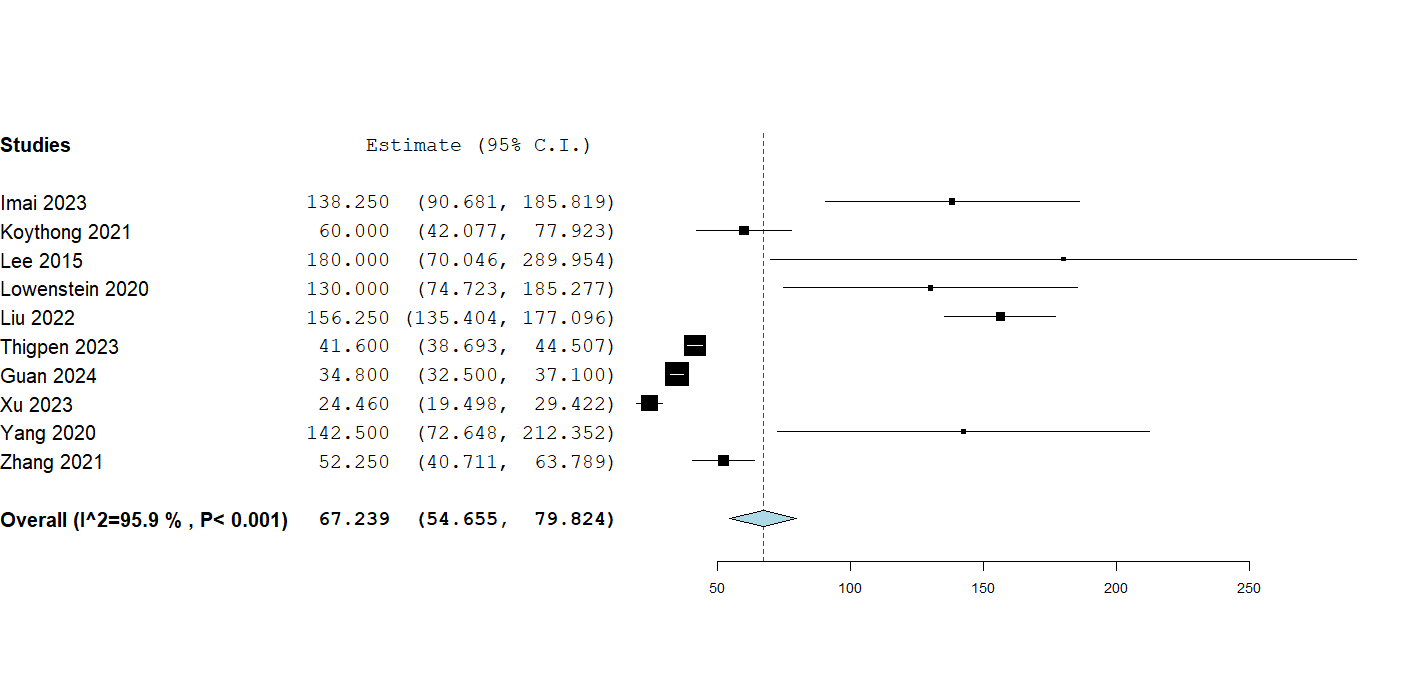


**Supplemental Figure S2:** Forest plot for the meta analysis of surgeon estimated blood loss (in mL.)


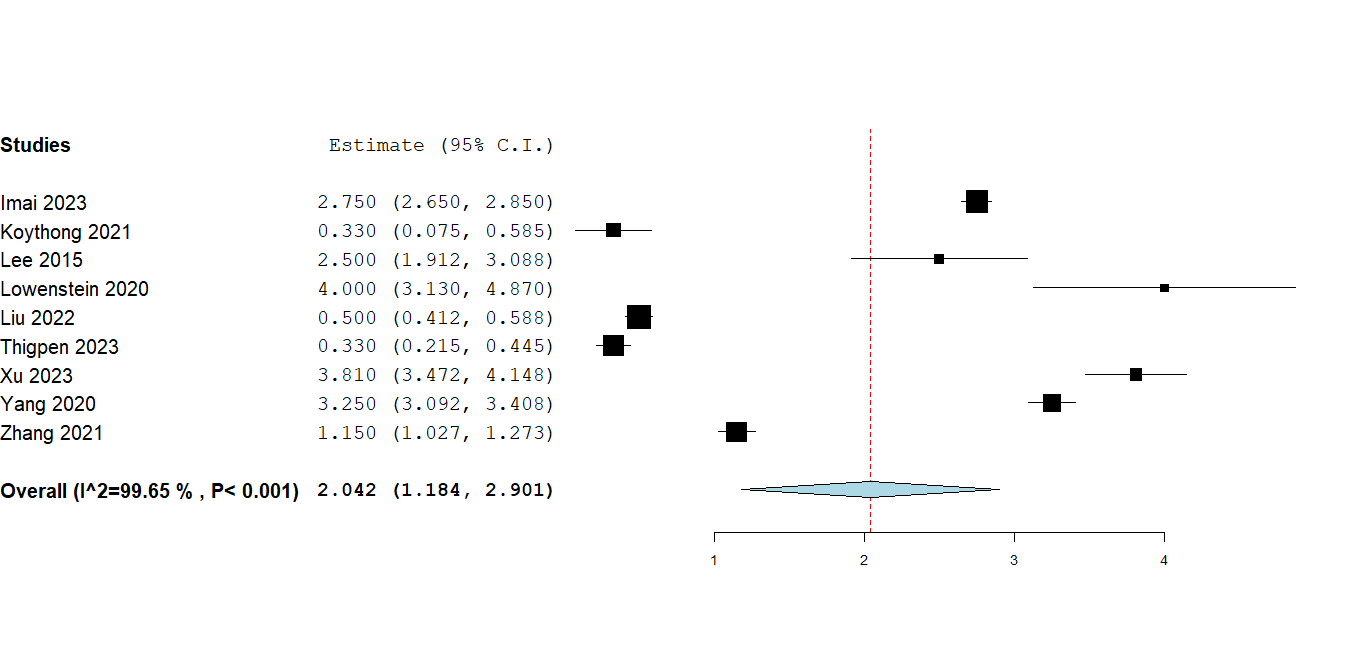


**Supplemental Figure S3:** Forest plot for the meta analysis of the length of hospital stay (in days.)


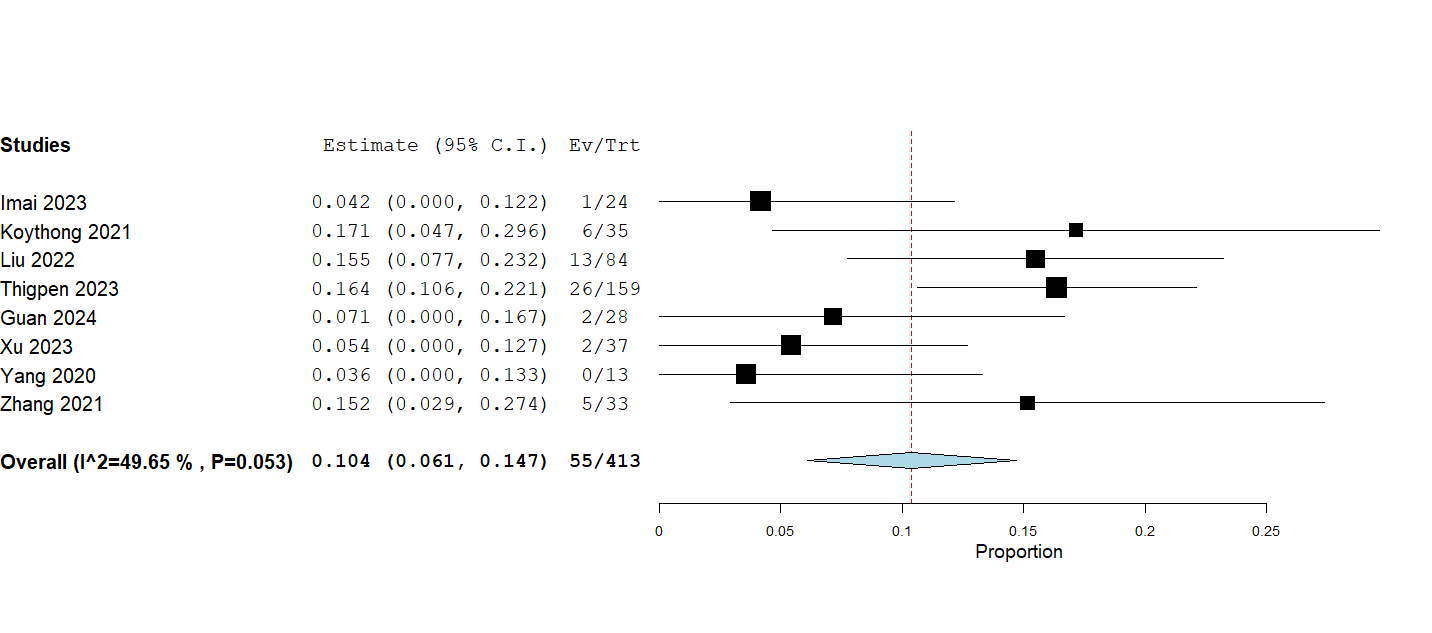


**Supplemental Figure S4:** Forest plot for the meta analysis of the rate of complications.


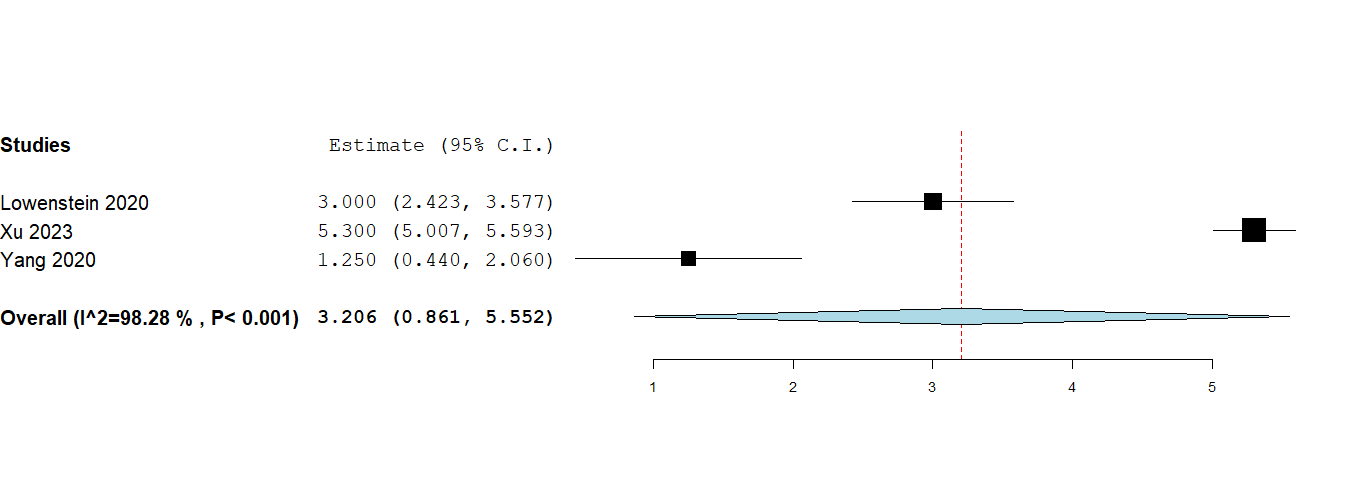


**Supplemental Figure S5:** Forest plot for the meta analysis of the VAS pain score at 24 hours postop.


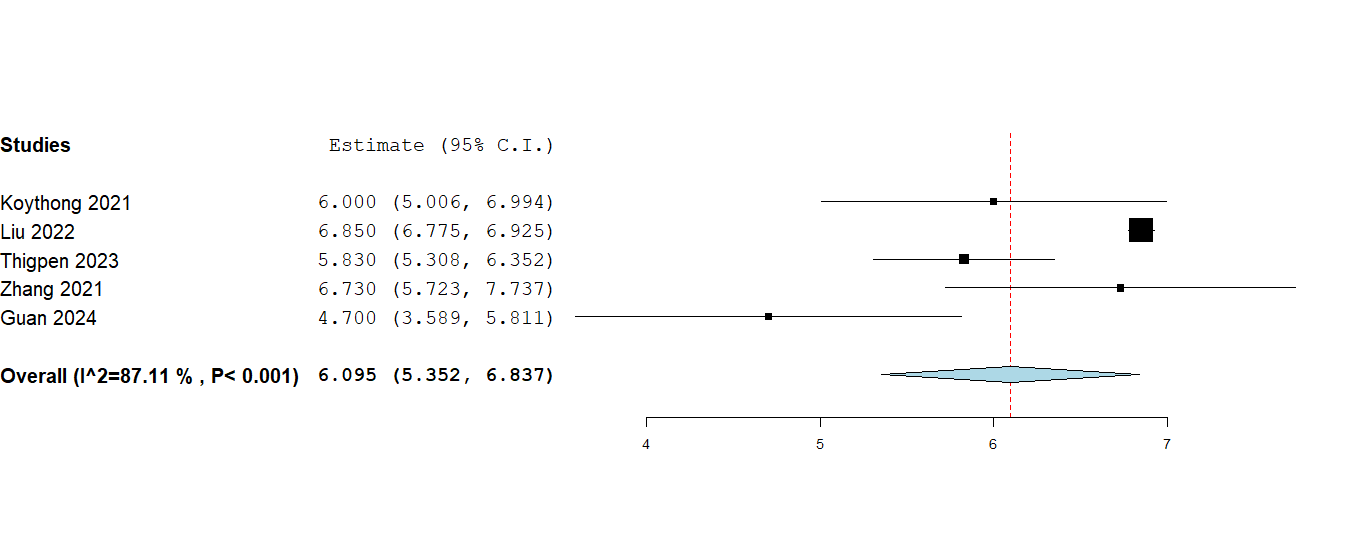

**Supplemental Figure S6:** Forest plot for the meta analysis of the VAS pain score at one week postop.


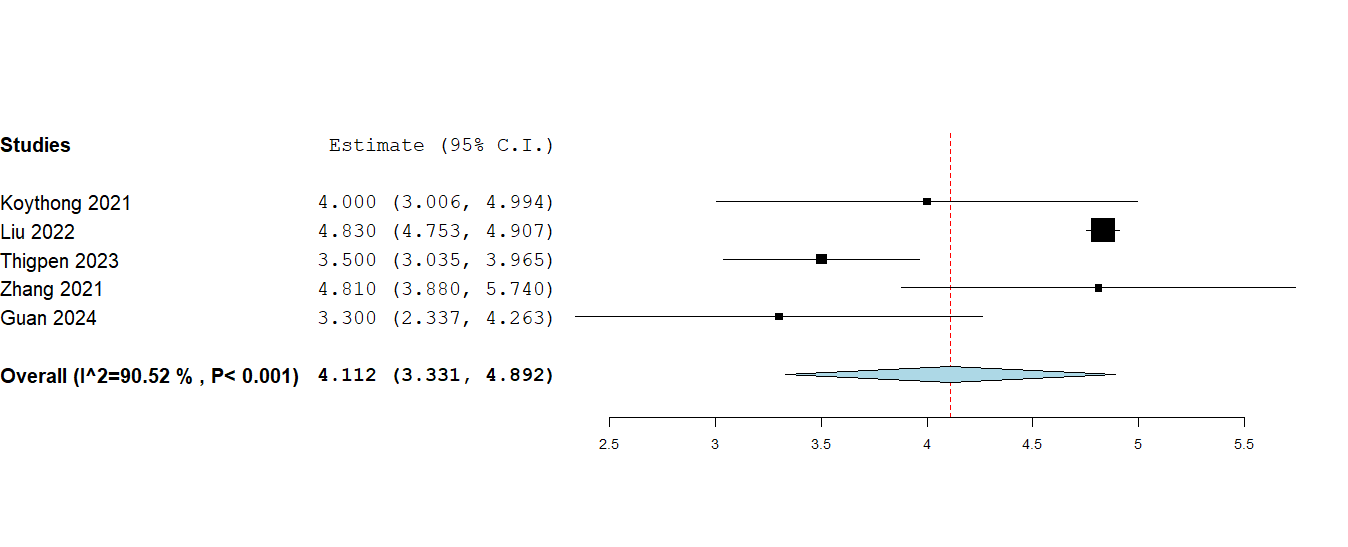


**Supplemental Figure S7:** Forest plot for the meta analysis of the VAS pain score at two weeks postop.


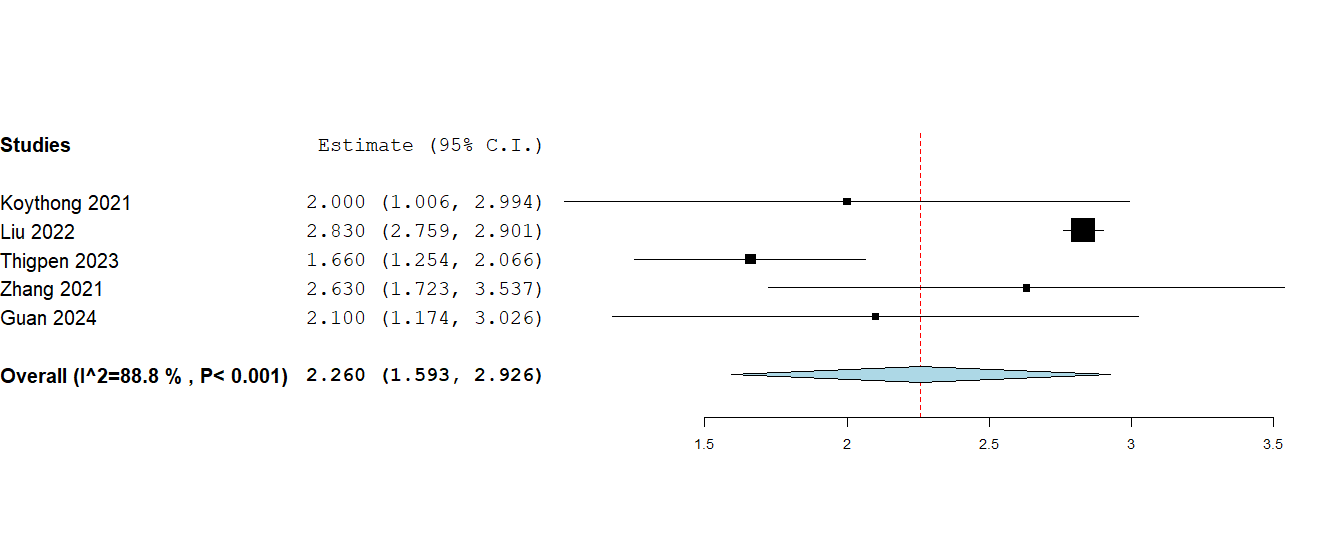


**Supplemental Figure S8:** Forest plot for the meta analysis of the VAS pain score at three weeks postop.


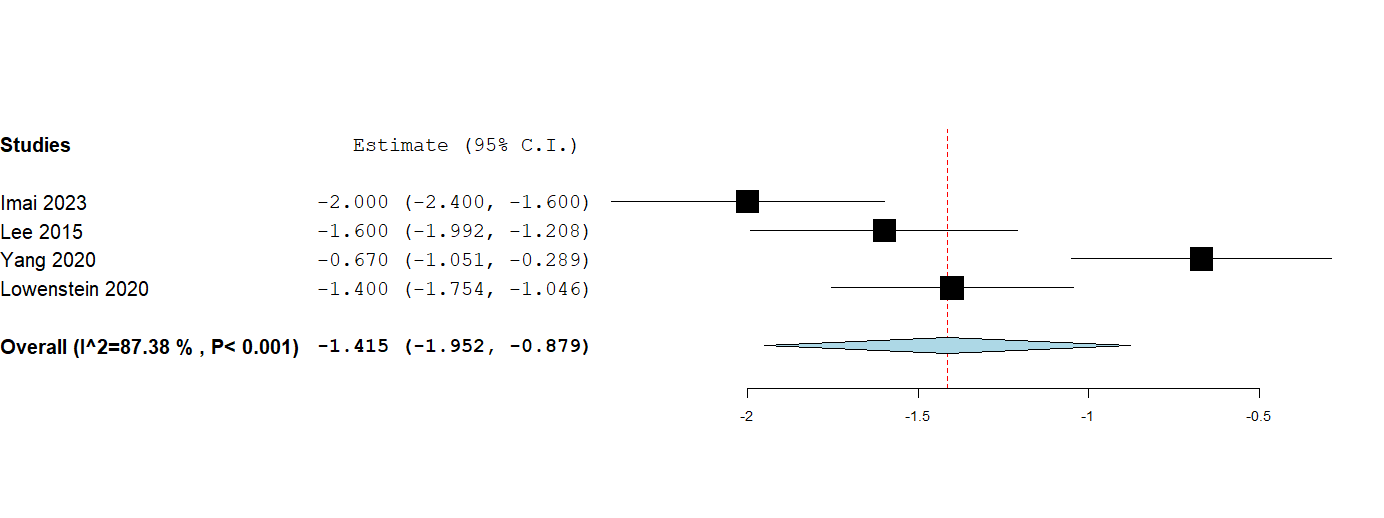


**Supplemental Figure S9:** Forest plot for the meta analysis of the change in hemoglobin level (in g/dL.)


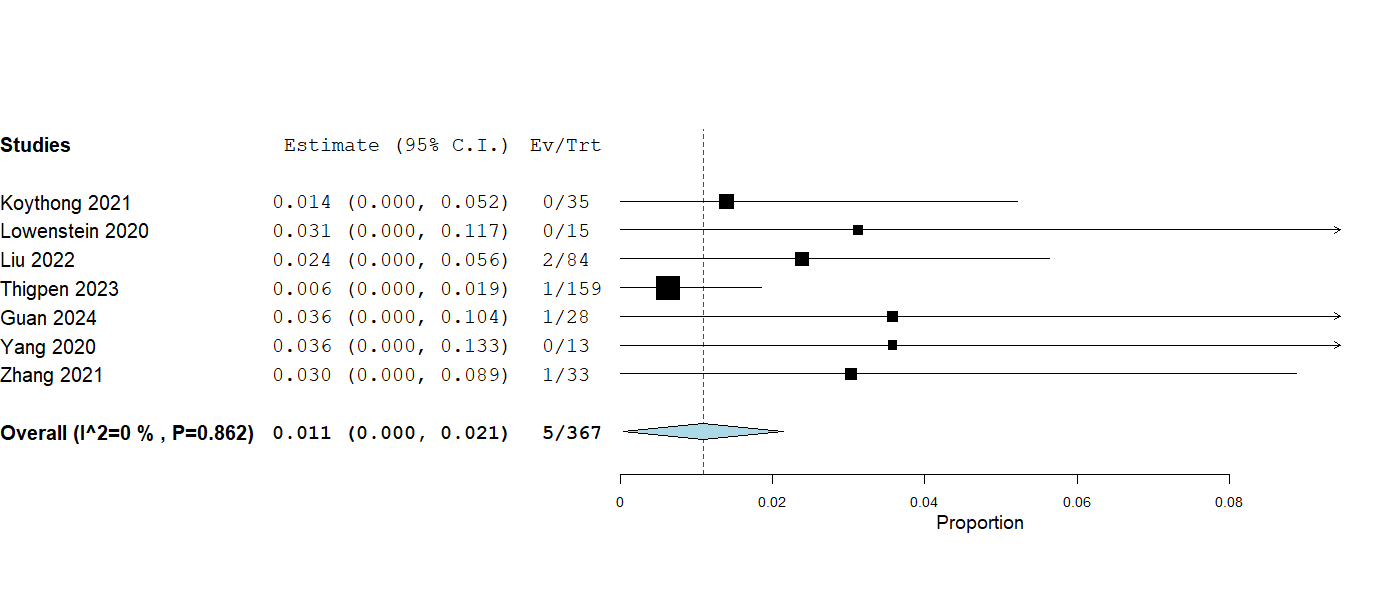


**Supplemental Figure S10:** Forest plot for the meta analysis of the rate of conversion to a different technique.
